# The impact of severe nephrotic syndrome on thyroid function, nutrition and coagulation

**DOI:** 10.1093/ckj/sfae280

**Published:** 2024-09-04

**Authors:** Anna Matyjek, Stanisław Niemczyk, Sławomir Literacki, Wojciech Fendler, Tomasz Rozmysłowicz, Andreas Kronbichler

**Affiliations:** Department of Internal Diseases, Nephrology and Dialysis, Military Institute of Medicine – National Research Institute, Warsaw, Poland; Department of Biostatistics and Translational Medicine, Medical University of Lodz, Lodz, Poland; Department of Internal Medicine IV, Nephrology and Hypertension, Medical University Innsbruck, Innsbruck, Austria; Department of Internal Diseases, Nephrology and Dialysis, Military Institute of Medicine – National Research Institute, Warsaw, Poland; Department of Laboratory Diagnostics, Military Institute of Medicine – National Research Institute, Warsaw, Poland; Department of Biostatistics and Translational Medicine, Medical University of Lodz, Lodz, Poland; Department of Pathology and Laboratory Medicine, University of Pennsylvania, Philadelphia, PA, USA; Department of Internal Medicine IV, Nephrology and Hypertension, Medical University Innsbruck, Innsbruck, Austria

**Keywords:** coagulation, hypothyroidism, nephrotic syndrome, nutrition, thyroid dysfunction

## Abstract

**Background:**

Nephrotic syndrome (NS) is characterized by urinary loss of proteins, including hormones and their carrier proteins, potentially resulting in endocrine disorders. This study aimed to assess thyroid dysfunction frequency and potential implications in NS.

**Methods:**

In this case–control study, patients with severe NS (serum albumin ≤2.5 g/dl) and controls without proteinuria were evaluated for thyroid, haemostatic and nutritional parameters, including body composition.

**Results:**

A total of 42 nephrotic and 40 non-proteinuric patients were enrolled. The NS group showed higher thyroid-stimulating hormone and lower free hormones, corresponding to a higher frequency of both euthyroid sick syndrome {ESS; 36% versus 5%; odds ratio [OR] 10.6 [95% confidence interval (CI) 2.2–50.0]} and hypothyroidism [31% versus 5%; OR 8.5 (95% CI 1.8–40.7)] compared with the control group. Levothyroxine supplementation was required for 11 NS patients (26% of the NS group). In addition, compared with control individuals, NS patients exhibited lower lean tissue mass and a trend towards hypercoagulability, which was evidenced by higher levels of most coagulation factors and fibrinolysis inhibitors, and reduced endogenous anticoagulants activities. Furthermore, NS patients with ESS presented with a 10.4 kg (95% CI −18.68 to −2.12) lower lean tissue mass. Those with hypothyroidism had significantly reduced activity of coagulation factor X [by −30% (95% CI −47 to −13)] and protein S [by −27% (95% CI −41 to −13)] compared with euthyroid NS individuals.

**Conclusions:**

Thyroid dysfunction is common in severe NS, often necessitating levothyroxine supplementation, which supports routine thyroid workup. A potential link between thyroid, nutritional and coagulation disorders in NS requires further investigation.

## Graphical Abstract

**Graphical Abstract:**
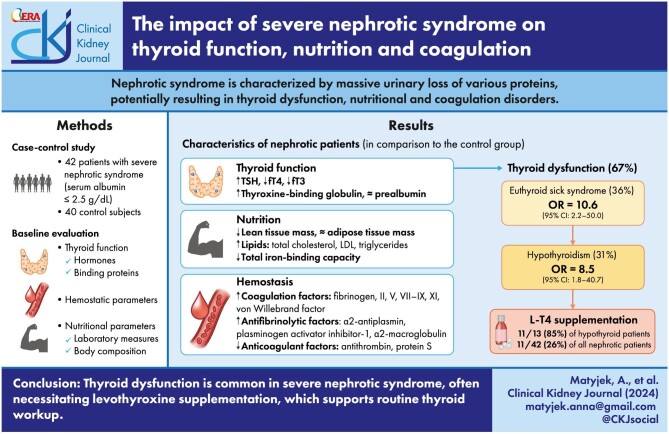

KEY LEARNING POINTS **What was known:**

- Patients with nephrotic syndrome (NS) have a substantial loss of thyroid hormones and carrier proteins during episodes of active nephrosis.
- Little is known about whether changes in thyroid hormones impact metabolic factors and the coagulation system.

**This study adds:**

- A substantial proportion of patients develop hypothyroidism (31%) and euthyroid sick syndrome (ESS; 36%) after onset of NS and ≈26% may require levothyroxine supplementation.
- Patients with severe NS, particularly those with ESS, have diminished lean tissue mass and exhibit significant coagulation abnormalities, which are more pronounced when overt hypothyroidism is present.
- The pathomechanism of thyroid hormones and haemostasis dysregulation appears to be more complex than resulting solely from the leakage of carrier proteins into urine.

**Potential impact:**

- It seems pivotal to check for thyroid function abnormalities in patients with severe NS.
- It seems that thyroid function may additionally impact nutrition and haemostasis in severe NS, therefore longitudinal studies are warranted to evaluate its clinical significance.

## INTRODUCTION

Nephrotic syndrome (NS) is a clinical condition caused by various glomerular diseases, both primary and secondary. It is characterized by massive loss of plasma proteins in the urine, resulting in hypoalbuminaemia, hyperlipidaemia and oedema. Although albumin is the dominant fraction of plasma proteins, and therefore experiences the greatest deficiency, the urinary loss disturbs the homeostasis of other biologically active substances, including peptide hormones and their binding proteins. Among the endocrine consequences of NS, thyroid dysfunction is most often described [1].

Thyroid hormones, both thyroxine (T4) and triiodothyronine (T3), are bound to the carrier proteins in the plasma at >99%. The main carrier protein is thyroxine-binding globulin (54 kDa), which binds >75% of thyroid hormones, followed by prealbumin (also called transthyretin; 55 kDa) and albumin (67 kDa)—each binding approximately 10–15% [2, 3]. Their low molecular weight facilitates their leakage into urine in NS. This, in turn, results in the loss of bound fractions of T4 and T3 [4–7], leading to a decrease in total blood T4 and T3 levels. It is believed that the concentrations of free fractions (fT4 and fT3) are maintained within the normal ranges in the majority of patients [1]. However, massive urinary loss of thyroid hormones along with their carrier proteins may exceed compensatory capabilities, resulting in either free hormones deficiency only, so called euthyroid sick syndrome (ESS), or elevation of serum thyroid stimulating hormone (TSH), and consequently hypothyroidism, even in patients without primary thyroid disease [8]. A pre-existing thyroid disorder facilitates the development of thyroid dysfunction in NS due reduced thyroid reserve [9]. The reversibility of abnormalities in the thyroid hormone profile after achieving NS remission confirms these pathophysiological connections [10, 11].

The clinical relevance of thyroid dysfunction in NS remains unclear. It is believed that due to its transient nature, hormone replacement therapy is generally not needed. However, it is unknown if thyroid dysfunction affects metabolic or haemostatic pathways, potentially worsening these known complications of NS [12, 13]. The implications of thyroid dysfunction on nutrition and coagulation are well documented in the general population [14–16], but their impact in the context of NS is still not fully understood. Recognizing this gap, we aimed to investigate the frequency of thyroid dysfunction and its relationship with nutritional and haemostatic parameters in severe NS.

## MATERIALS AND METHODS

### Study design and participants

This is a case–control study that included a previously described group of 42 patients with severe NS (the NS group) [17] and 40 individuals without proteinuria (the control group). The control group consisted of 23 patients from a previous report [17] and 17 patients additionally enrolled for this study. Details of the original study design have been reported elsewhere [17]. Briefly, patients with an episode of non-diabetic NS, either with a new diagnosis or a relapse, with serum albumin ≤2.5 mg/dl (reference range 3.8–5.2 g/dl) and an estimated glomerular filtration rate (eGFR) ≥30 ml/min/1.73 m^2^ were recruited to the NS group. Individuals without proteinuria and similar in terms of age, sex, height, weight and kidney function served as the control group.

### Data collection

Data including demographics, NS course, as well as thyroid, haemostatic and nutritional parameters were collected at baseline, i.e. at diagnosis or relapse of NS (in the NS group) or at enrolment (in the control group).

Thyroid workup included TSH, fT4, fT3 and serum concentrations of carrier proteins: thyroxine-binding globulin (TBG ELISA Kit, Wuhan EIAab Science, Wuhan, China), prealbumin (Roche Diagnostics, Rotkreuz, Switzerland) and serum albumin (bromocresol green–based Albumin Gen.2 Test, Roche Diagnostics).

Thyroid dysfunction was defined as the presence of one of the following: ESS or subclinical or overt hypothyroidism. ESS was defined as an isolated decrease in fT3 and/or fT4 (<3.2 pmol/l and <12 pmol/l, respectively) and hypothyroidism as an increased TSH level (>4.2 µIU/ml) along with in-range (subclinical) or reduced (overt) fT4 concentration.

Baseline values of nutritional and haemostatic parameters were collected to evaluate their relationships with thyroid function in severe NS. Nutritional measures included serum lipid levels, total iron-binding capacity and body composition measured by bioimpedance spectroscopy using the Body Composition Monitor (Fresenius Medical Care, Bad Homburg, Germany). The evaluation of haemostasis included measurement of plasma coagulation and fibrinolysis factors (fibrinogen concentration; activity of factors II, V, VII–XII and von Willebrand—using the von Willebrand factor Ristocetin cofactor assay; and plasminogen) and the activity of inhibitors of both coagulation (antithrombin, protein C and protein S using a free antigen assay) and fibrinolysis [α2-antiplasmin and α2-macroglobulin (Siemens Healthcare Diagnostics Products, Marburg, Germany) and plasminogen activator inhibitor-1 (PAI-1 ELISA Kit, Wuhan EIAab Science, Wuhan, China). Unless specified otherwise, coagulation tests were performed using the ACL TOP 500 CTS analyzer and HemosIL reagents (Instrumentation Laboratory, Bedford, MA, USA).

The study was approved by the local bioethics committee (approval 63/WIM/2015). All patients signed an informed consent.

### Statistical analysis

Continuous variables were presented as mean with standard deviation (SD) for normally distributed data (tested with the Shapiro–Wilk test) and median with 25–75% interquartile range (IQR) for non-normally distributed data and numbers with percentages for qualitative variables.

The comparison between the NS and control groups was conducted using the unpaired *t*-test for normally distributed variables and the Mann-Whitney U test for non-normally distributed continuous variables. The Fisher's exact test or χ^2^ test with the Yates's continuity correction was used as appropriate for categorical variables. Relationships between thyroid hormones, carrier proteins, indicators of the NS course and haemostatic and nutritional parameters were evaluated using Spearman's rank correlation coefficients and presented as a correlation matrix.

The comparison between euthyroid, ESS and hypothyroid nephrotic patients was performed using analysis of variance (ANOVA), Kruskal–Wallis test or χ^2^ test, as appropriate.

The differences between groups (with *P* < .05) in terms of nutritional and coagulation parameters were further analysed using generalized linear models. These analyses aimed to ascertain the impact of thyroid dysfunction on nutrition and haemostasis, while accounting for potential confounding factors such as indicators of the NS course (serum albumin, proteinuria, eGFR), as well as known factors affecting nutrition (sex, age, height, weight and eGFR). For this purpose, the univariate models were constructed, followed by the development of multivariate models consisting of variables showing *P*-values <.10 in the univariate analysis. The results of multivariate analysis were presented as standardized coefficients (β) with 95% confidence intervals (CIs) in the forest plots and coefficients with standard errors (provided in the supplementary materials). The quality of each model was assessed using corrected coefficients of determination (*R*^2^ referring to the percentage of variation explained by the model).

Data analysis was preformed using Statistica version 13.3 (Tibco Software, Palo Alto, CA, USA), with the use of two-tailed tests and *P*-values <.05 considered statistically significant.

## RESULTS

A total of 42 patients (29 males and 13 females) with severe NS with an average proteinuria of 8.9 g/24 h (the NS group) and 40 individuals without proteinuria (the control group) were enrolled. As reported previously [17], the leading causes of NS were minimal change disease (MCD) and primary focal segmental glomerulosclerosis (FSGS) (22 cases), followed by membranous nephropathy (MN; 11 cases). A median duration of NS symptoms prior to the study entry was <1 month and 12 patients were on immunosuppressive treatment at the time of enrolment. Past medical history was remarkable for well-controlled Hashimoto thyroiditis in 10% of NS patients and in none of the non-proteinuric individuals. There was no history of other thyroid disorders in any of the remaining patients.

### Thyroid function

The NS group presented with higher serum TSH (2.68 versus 1.37 µIU/ml, *P* < .001) and lower free hormone levels [fT4 (12.46 versus 17.07 pmol/l, *P* < .001) and fT3 (3.34 versus 4.59 pmol/l, *P* < .001)] than the control group (Table 1). Thyroid dysfunction was diagnosed in 67% of patients with severe NS: ESS in 15 (36%) and hypothyroidism in 13 (31%) patients, with overt in 8 (19%) and subclinial in 5 (12%). Newly diagnosed thyroid abnormalities were observed more frequently in NS than in the control group: ESS 10.6 times more frequently [36% versus 5%; OR 10.6 (95% CI 2.2–50.0)], hypothyroidism 8.5 times [31% versus 5%; OR 8.5 (95% CI 1.8–40.7)] and any thyroid dysfunction 18 times [67% versus 10%; OR 18.0 (95% CI 5.3–60.7)].

**Table 1:** Baseline characteristics of the groups.

Variable	NS group (*n* = 42)	Control group (*n* = 40)	*P*-value
**Demography and anthropometry**			
Sex, *n* (%)			
Male	29 (69.0)	29 (72.5)	
Female	13 (31.0)	11 (27.5)	.919
Age (years), mean ± SD	49 ± 20	47 ± 20	.691
Height (cm), mean ± SD	170 ± 10	173 ± 10	.134
Weight (kg), mean ± SD	81 ± 17	78 ± 15	.276
**NS course**			
Serum albumin (g/dl), median (IQR)	2.1 (1.6–2.4)	4.6 (4.3–5.0)	**<.001**
Proteinuria (g/24 h), median (IQR)	8.9 (6.0–12.9)	NA	NA
Duration of nephrotic syndrome (months), median (IQR)	0.9 (0.25–3.0)	NA	NA
Histology, *n* (%)			
MCD/FSGS	22 (52.4)	NA	NA
MN	11 (26.2)		
Other or unknown	9 (21.4)		
Serum creatinine (mg/dl), median (IQR)	1.1 (0.8–1.6)	1.1 (0.8–1.3)	.278
eGFR (ml/min/1.73 m^2^), median (IQR)	70 (42–105)	86 (61–102)	.290
**Prior history of thyroid disorder, *n* (%)**			
Hashimoto disease	4 (10)	0	.116
None	38 (90)	40 (100)	
**Newly diagnosed thyroid dysfunction, *n* (%)**			
Hypothyroidism	13 (31)	2 (5)	**<.001**
ESS	15 (36)	2 (5)	
None	14 (33)	36 (90)	
**Thyroid hormones and carrier proteins, median (IQR)**			
TSH (µIU/ml)	2.68 (1.35–4.96)	1.37 (0.96–1.73)	**<.001**
fT4 (pmol/l)	12.46 (10.66–15.42)	17.07 (15.22–18.24)	**<.001**
fT3 (pmol/l)	3.34 (2.53–3.81)	4.59 (3.84–4.93)	**<.001**
Thyroxine-binding globulin (ng/ml)	3.51 (2.65–4.58)	1.87 (1.00–3.09)	**<.001**
Prealbumin (mg/dl)	26 (21–30)	26 (22–30)	.683
**Nutrition**			
Lean tissue mass (kg), mean ± SD	38.3 ± 11.4	45.1 ± 10.7	**.007**
Adipose tissue mass (kg), median (IQR)	33.7 (28.7–43.9)	30.2 (22.1–41.0)	.138
Total cholesterol (mg/dl), mean ± SD	360 ± 124	167 ± 52	**<.001**
LDL (mg/dl), mean ± SD	253 ± 99	104 ± 45	**<.001**
Triglycerides (mg/dl), medina (IQR)	192 (149–299)	102 (82–160)	**<.001**
Total iron-binding capacity (µg/dl), mean ± SD	175 ± 38	298 ± 68	**<.001**
**Haemostasis**			
Fibrinogen (mg/dl), mean ± SD	661 ± 184	346 ± 167	**<.001**
Factor II (%), mean ± SD	115 ± 18	93 ± 12	**.030**
Factor V (%), mean ± SD	127 ± 48	91 ± 30	**.003**
Factor VII (%), mean ± SD	119 ± 34	96 ± 35	**.003**
Factor VIII (%), mean ± SD	171 ± 68	107 ± 44	**.006**
Factor IX (%), mean ± SD	149 ± 46	127 ± 49	**.036**
Factor X (%), mean ± SD	100 ± 24	93 ± 20	.131
Factor XI (%), mean ± SD	131 ± 38	103 ± 47	**.004**
Factor XII (%), mean ± SD	77 ± 37	115 ± 39	**<.001**
von Willebrand factor (%), median (IQR)	134 (118–288)	108 (89–119)	**<.001**
Protein S (%), mean ± SD	81 ± 22	96 ± 23	**.002**
Protein C (%), mean ± SD	228 ± 68	129 ± 27	**<.001**
Antithrombin (%), mean ± SD	80 ± 22	100 ± 14	**<.001**
Plasminogen (%), mean ± SD	95 ± 15	95 ± 14	.942
Plasminogen activator inhibitor-1 (ng/ml), median (IQR)	0.96 (0.47–1.66)	0.27 (0.06–0.70)	**<.001**
α2-antiplasmin (%), mean ± SD	113 ± 13	107 ± 9	**.013**
α2-macroglobulin (mg/dl), median (IQR)	364 (283–449)	174 (144–190)	**<.001**

Significant values in bold.

NA: not applicable.

There were no differences between patients with different histological diagnoses (Supplementary Table S1), nor with a new diagnosis versus relapse of NS in terms of occurrence of thyroid dysfunction, although relapsers tended to present with lower TSH (Supplementary Table S2).

Based on the thyroid workup results, 11 NS patients (9 without prior thyroid disorder and 2 with previously well-controlled Hashimoto disease) were recommended to start or adjust the dose of levothyroxine according to current guidelines (Supplementary Table S3).

### Carrier proteins

**Figure 1:**
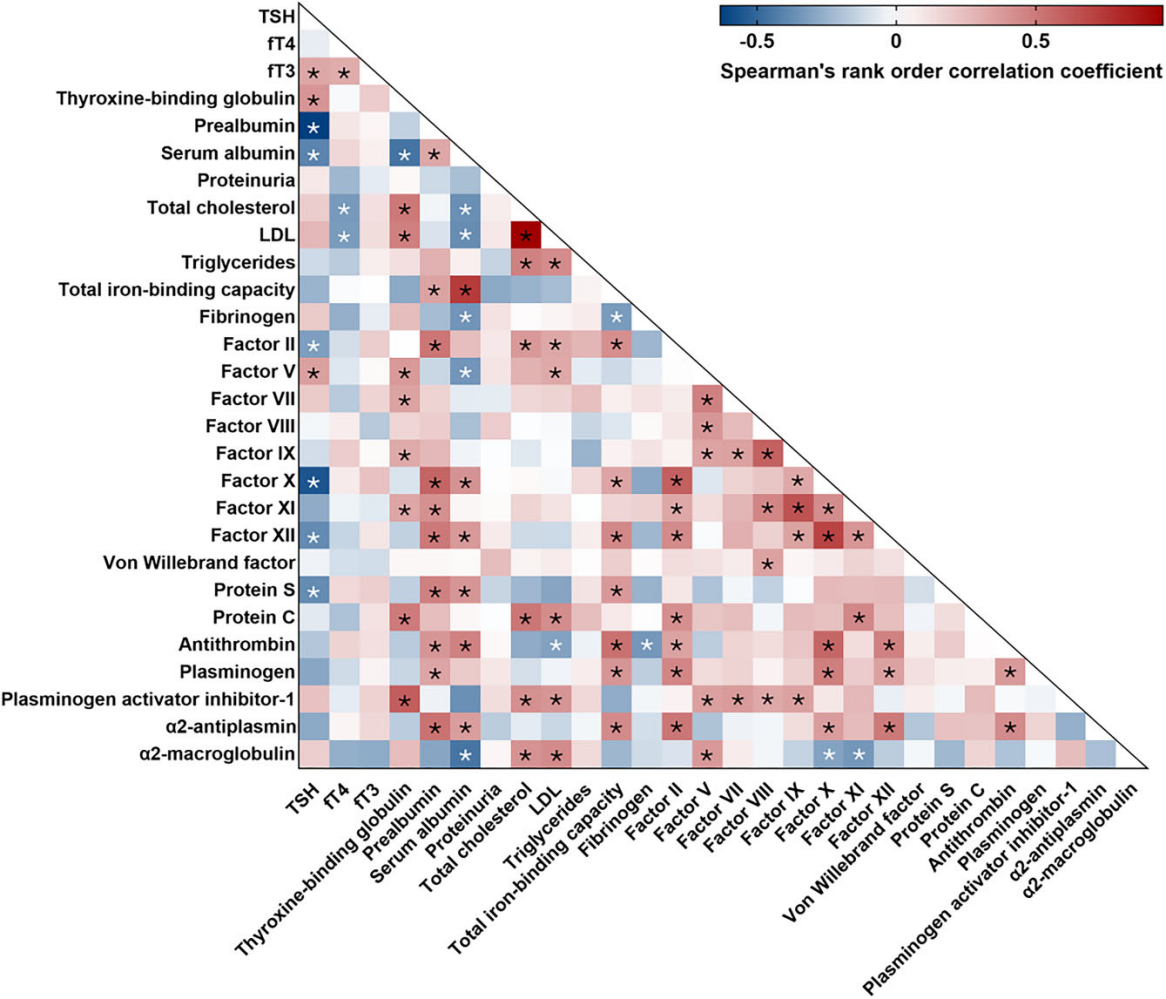
Heat map of correlations between thyroid hormones, carrier proteins and nutritional and coagulation parameters in severe NS. *Spearman's rank correlations of statistical significance (*P* *< *.05). The exact *R* and *P*-values are provided in Supplementary Table S2.

Nephrotic patients presented with higher thyroxin-binding globulin (3.51 versus 1.87 ng/ml; *P* < .001) but not prealbumin levels compared with the control group (Table 1). Both carrier proteins significantly correlated with TSH in NS (Fig. 1, Supplementary Table S4), however, their relationships displayed different directions: negative for prealbumin (*R* = −0.63, *P* < .001) and positive for thyroxine-binding globulin (*R* = 0.39, *P* = .010). Consistent with this, NS patients with hypothyroidism tended to display higher thyroxine-binding globulin levels than the ESS subgroup (*P* = .050) and showed significantly lower prealbumin levels than either ESS or euthyroid individuals (Table 2).

**Table 2:** Characteristics of nephrotic patients with euthyreosis, ESS and hypothyroidism.

Variable	Euthyreosis (*n* = 14)	ESS (*n* = 15)	Hypothyroidism (*n* = 13)	*P*-value
**Demography and anthropometry**				
Sex, *n* (%)				
Male	11 (78.6)	10 (66.7)	8 (61.5)	.614
Female	3 (21.4)	5 (33.3)	5 (38.5)	
Age (years), median (IQR)	55 (33–59)	56 (36–71)	31 (29–67)	.633
Height (cm), mean ± SD	173 ± 11	169 ± 9	168 ± 19	.438
Weight (kg), mean ± SD	86.6 ± 14.7	79.1 ± 15.7	78.1 ± 19.0	.343
**NS course**				
Serum albumin (g/dl), median (IQR)	2.3 (2.0–2.4)	2.1 (1.6–2.3)	2.0 (1.5–2.4)	.648
Proteinuria (g/24 h), mean ± SD	9.7 ± 4.2	10.2 ± 5.4	10.1 ± 5.9	.966
Duration of nephrotic syndrome (months), median (IQR)	1.0 (0.3–3.0)	2.0 (0.5–4.0)	0.8 (0.3–2.0)	.246
Histology, *n* (%)				
MCD/FSGS	7 (50)	8 (53)	7 (54)	.177
MN	6 (43)	4 (27)	1 (8)	
Other or unknown	1 (7)	3 (20)	5 (38)	
Serum creatinine (mg/dl), median (IQR)	1.1 (0.9–1.5)	1.1 (0.7–1.7)	1.4 (0.8–1.6)	.913
eGFR (ml/min/1.73 m^2^), median (IQR)	81 (52–103)	60 (40–109)	54 (42–105)	.967
**Prior history of thyroid disorder, *n* (%)**				
Hashimoto disease	0	2 (13.3)	2 (15.4)	
None	14 (100)	13 (86.7)	11 (84.6)	.326
**Thyroid hormones and carrier proteins**				
TSH (µIU/ml)^b^,^c^, median (IQR)	2.33 (1.34–2.93)	1.48 (1.05–2.05)	5.77 (5.02–6.44)	**<.001**
fT4 (pmol/l), median (IQR)	14.0 (12.6–16.3)	11.0 (10.6–14.6)	11.0 (9.3–15.4)	.075
fT3 (pmol/l)^a^,^c^, median (IQR)	3.7 (3.5–4.1)	2.3 (2.0–2.9)	3.4 (3.0–3.9)	**<.001**
Thyroxine-binding globulin (ng/ml), mean ± SD	3.75 ± 1.95	2.95 ± 1.01	4.41 ± 1.68	.061
Prealbumin (mg/dl)^b^,^c^, mean ± SD	29.43 ± 6.47	27.20 ± 7.44	19.00 ± 5.12	**<.001**
**Nutrition**				
Lean tissue mass (kg)^a^, mean ± SD	44.9 ± 12.7	34.5 ± 8.8	35.7 ± 10.0	**.024**
Adipose tissue mass (kg), median (IQR)	41.7 (31.3–45.4)	33.8 (28.5–43.9)	30.3 (29.7–34.3)	.533
Total cholesterol (mg/dl), median (IQR)	3.3 (2.4–4.9)	4.7 (2.1–5.5)	5.8 (3.4–7.1)	.102
LDL (mg/dl), mean ± SD	352 ± 92	347 ± 128	383 ± 153	.726
Triglycerides (mg/dl), mean ± SD	248 ± 72	235 ± 105	278 ± 119	.518
Total iron-binding capacity (µg/dl), mean ± SD	211 (149–253)	226 (161–344)	167 (128–209)	.526
**Haemostasis**				
Fibrinogen (mg/dl), mean ± SD	630 ± 115	621 ± 197	741 ± 215	.167
Factor II (%), mean ± SD	120 ± 19	117 ± 16	107 ± 16	.134
Factor V (%), mean ± SD	118 ± 56	121 ± 46	143 ± 41	.335
Factor VII (%), mean ± SD	117 ± 34	117 ± 38	124 ± 29	.829
Factor VIII (%), mean ± SD	161 ± 79	170 ± 55	183 ± 74	.716
Factor IX (%), mean ± SD	161 ± 50	141 ± 51	146 ± 34	.486
Factor X (%)^b^, mean ± SD	114 ± 24	101 ± 22	84 ± 18	**.004**
Factor XI (%), mean ± SD	147 ± 41	125 ± 38	120 ± 30	.151
Factor XII (%), mean ± SD	88 ± 44	78 ± 33	65 ± 32	.267
von Willebrand factor (%), median (IQR)	133 (118–240)	119 (117–330)	220 (119–297)	.527
Protein S (%)^b^, mean ± SD	95 ± 20	78 ± 22	68 ± 16	**.004**
Protein C (%), mean ± SD	259 ± 81	217 ± 36	207 ± 74	.105
Antithrombin (%), mean ± SD	82 ± 16	86 ± 21	72 ± 27	.264
Plasminogen (%), mean ± SD	99 ± 14	98 ± 12	88 ± 18	.123
Plasminogen activator inhibitor-1 (ng/ml), median (IQR)	1.28 (0.35–1.66)	0.74 (0.31–1.78)	1.06 (0.68–1.35)	.571
α2-antiplasmin (%), mean ± SD	116 ± 11	114 ± 14	110 ± 14	.545
α2-macroglobulin (mg/dl), mean ± SD	323 ± 116	420 ± 117	413 ± 147	.093

Significant values in bold.

Footnotes indicate statistically significant results (*P* < .05) of the pairwise comparisons of the following groups: ^a^euthyreosis versus ESS; ^b^euthyreosis versus hypothyroidism; ^c^ESS versus hypothyroidism.

It should be noted that although serum albumin correlated with both, TSH (*R* = −0.41, *P* = .007) and carrier proteins (*R* = −0.46, *P* = .002 for thyroxin-binding protein; *R* = 0.32, *P* = .036 for prealbumin) (Fig. 1, Supplementary Table S4), no differences between euthyroid, ESS and hypothyroid subgroups were observed in terms of serum albumin or any other indicator of NS severity (Table 2).

### Nutrition

**Figure 2:**
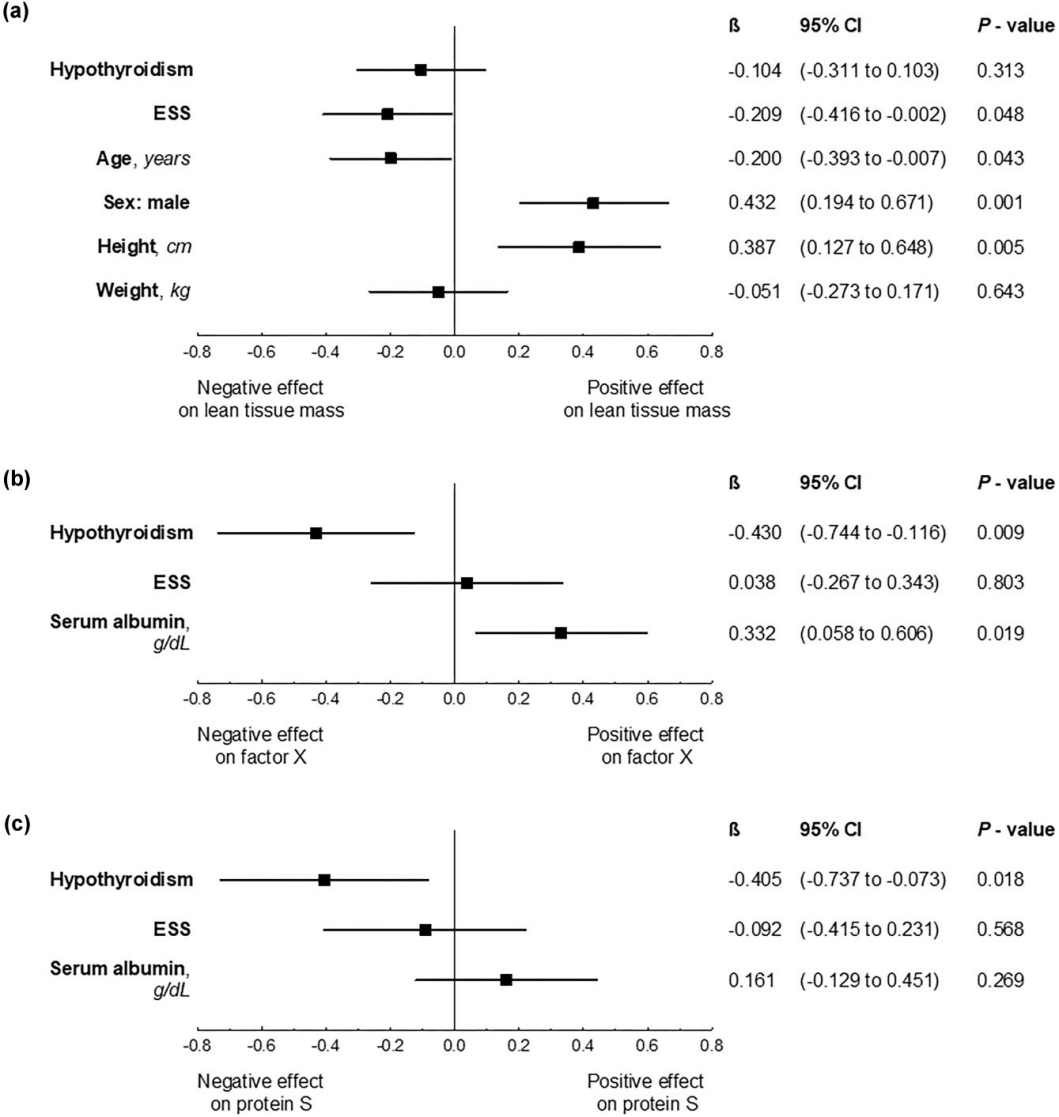
The effect of thyroid dysfunction on **(a)** lean tissue mass, **(b)** factor X and **(c)** free protein S activity in severe NS in the multivariate models. β: standardized coefficient of the linear regression model. A subgroup of patients with NS and euthyroidism serves as the reference group.

Body composition analysis revealed lower lean tissue mass in patients with severe NS compared with the control group [mean difference −6.80 kg (95% CI −11.66 to −1.94)], despite similar demographic and anthropometric features and similar adipose tissue mass (Table 1). In addition, nephrotic patients with ESS had significantly lower lean tissue mass compared with euthyroid nephrotic individuals [mean difference −10.4 kg (95% CI −18.68 to −2.12)] (Table 2). This negative relationship between ESS and lean tissue mass in severe NS was still noticeable after adjustment for known factors affecting nutritional status (Fig. 2a, Supplementary Table S5).

In terms of laboratory parameters of nutrition, the NS group presented with higher lipid levels [all: total cholesterol, low-density lipoprotein (LDL) and triglycerides] and lower total iron-binding capacity than the control group (Table 1). Although there were moderate negative correlations between fT4 and both total cholesterol (*R* = −0.34, *P* = .030) and LDL (*R* = −0.33, *P* = .033) (Fig. 1, Supplementary Table S4), no differences in lipid levels were found between patients with NS and different thyroid hormone profiles. Similarly, total iron-binding capacity did not differ between these subgroups (Table 2).

### Haemostasis

Compared with the control group, the NS group presented with significantly higher activities of most plasma coagulation factors, including fibrinogen; factors II, V, VII–XII and von Willebrand factor; and, in addition, higher levels of inhibitors of fibrinolysis: α2-antiplasmin, α2-macroglobulin and plasminogen activator inhibitor-1. In contrast, antithrombin and factor XII activity were found lower in NS than in patients without proteinuria (Table 1). Among nephrotic patients, relapsers presented with higher activities of factors X–XII than patients with newly diagnosed NS (Supplementary Table S2); of note, no significant differences were noted between MCD/FSGS and MN patients in terms of haemostatic factors (Supplementary Table S1).

TSH showed negative correlations with factors II (*R* = −0.32, *P* = .039), X (*R* = −0.56, *P* < .001), XII (*R* = −0.39, *P* = .012) and protein S (*R* = −0.38, *P* = .012), while a positive relationship with factor V (*R* = 0.35, *P* = .022) in severe NS was observed (Fig. 1, Supplementary Table S4).

Consistently, hypothyroid NS patients presented with lower activity of both factor X [mean difference −30% (95% CI −47 to −13)] and protein S [mean difference −27% (95% CI −41 to −13)] compared with euthyroid NS patients. The effects of hypothyroidism on factor X and protein S activities were still prominent after adjustment for serum albumin as an indicator of NS severity (Fig. 2b and c, Supplementary Tables S6 and S7).

## DISCUSSION

### Frequency, diagnostics and management of thyroid dysfunction in NS

The study revealed abnormalities in thyroid hormone profiles in the majority of patients (67%) at presentation of severe NS with serum albumin ≤2.5 g/dl. These findings align with a prior report from a large retrospective study reporting a frequency of 80% in patients with varying degrees of proteinuria. The distribution pattern observed in our study mirrored the previously reported one, with ESS being the most prevalent disorder, followed by subclinical hypothyroidism [18]. Conversely, the paediatric population with NS has exhibited a different distribution, with hypothyroidism clearly predominating [10].

We found hypothyroidism was 8.5-fold more likely to be diagnosed in NS than in the control group. Despite the small group size, and thus the low precision of the estimate (wide confidence bounds), our results are similar to those reported in a large Dutch study. Thyroid dysfunction, including both ESS and hypothyroidism, was found to occur 7.8 times more often in nephrotic patients than in matched controls from the general Dutch population (15.7% versus 2.3%) [19]. Notably, in 11 of 13 hypothyroid nephrotic patients from our study (26% of the whole NS group), levothyroxine initiation or dose adjustment was required, aligning with the treatment recommendations outlined by the American and European Thyroid Associations [20, 21], even though most of them had no prior history of thyroid disease. Thus our study provides a rationale for advocating routine thyroid function testing in severe NS in adults, despite the absence of such a recommendation in the Kidney Disease: Improving Global Outcomes guidelines [22]. Of note, paediatric guidelines endorse routine assessments at diagnosis and at least every 12 months in cases of persistent proteinuria [23].

Moreover, the revealed associations between thyroid function and nutritional and coagulation disorders should be considered in the discussion of a potential clinical significance of thyroid dysfunction in NS.

### Thyroid dysfunction and nutritional status in NS

Both NS and thyroid function influence nutritional status. We found lean tissue mass lower in the NS than in the control group, suggesting a potential impact of urinary loss of proteins on muscle mass. Animal models have demonstrated increased protein turnover and albumin synthesis in NS [24–26], likely utilizing amino acids from muscle catabolism. Additionally, the significantly lower lean tissue mass in ESS seems to parallel the well-documented link between ESS and malnutrition in the general population [27, 28]. This is pathophysiologically explicable, as T3 regulates gene expression in muscle cells, influencing pathways related to muscle development and repair, and overall cell function, including metabolism and energy expenditure [29]. However, the causal relationship between thyroid abnormalities and lean tissue deficit in severe NS remains speculative.

Notably, unlike hypothyroidism's effect on fat gain or hyperlipidaemia in the general population, no significant relationship between thyroid dysfunction in NS and adipose tissue mass or lipid levels was detected. This discrepancy could be attributed to the presumed short duration of thyroid dysfunction likely insufficient to impact adipose tissue and to the limited effect of thyroid dysfunction on lipid levels compared to the direct impact of NS. However, the dynamics of catabolism-synthesis processes in NS and their long-term impact on nutrition have not been thoroughly investigated and still remain unclear.

### Thyroid dysfunction and haemostasis in NS

More is known about coagulation disorders in NS. Venous thromboembolism is one of its most threatening complications, occurring primarily in the first months [30–32], and its estimated risk in the first year after diagnosis is 7.11 times higher than in the general population [33]. Hypothyroidism is also a known trigger for thromboembolic complications, increasing the risk from 1.27- to 1.82-fold compared with euthyroid individuals across all age groups [15, 16, 34]. However, the cumulative effect of both entities on haemostatic parameters has not been investigated so far.

A prothrombotic state is considered as the main factor contributing to thromboembolic events in NS. Numerous studies [12, 31], including ours, have revealed a trend towards hypercoagulability. Compared with the control group, our NS group presented with significantly higher levels of most coagulation factors (II, V, VII–IX, XI, XII, von Willebrand factor) and fibrinolysis inhibitors (plasminogen activator inhibitor-1, α2-antiplasmin, α2-macroglobulin) were found, while activities of essential endogenous anticoagulants (antithrombin, protein S) were decreased.

The serum albumin level appears to be the best marker of severity of NS and therefore a crucial predictor of the intensity of the prothrombotic state and venous thromboembolism risk. A level of 2.5–3.0 g/dl increases the hazard by 2.25 times and a level <2.5 g/dl increases it by 2.79 times compared with an in-range albumin concentration (>4.0 g/dl) [35]. Although smaller studies have also indicated the significance of proteinuria in predicting these complications in patients with membranous nephropathy [32, 36], the largest retrospective study in this glomerular disease did not confirm the value of proteinuria as a potential risk factor [30]. A serum albumin level <2.8 g/dl was the only predictor and was associated with 2.13-fold increased risk of venous thromboembolism [30]. This finding is consistent with the significant correlations between albumin and numerous haemostatic parameters in our study and the lack of such relationships for proteinuria. It may result from the generally poor reproducibility of 24-h urine collection results due to the cumbersome nature of the procedure and consequently limited patient compliance [37, 38].

Although the highest risk of thromboembolic complications is associated with a histological diagnosis of membranous nephropathy—a 10.8-fold higher risk compared with immunoglobulin A nephropathy (showing the lowest risk among primary glomerular diseases) [39]—the exact pathomechanism remains unknown and seems attributable to the nature of the disease itself. Our study also did not reveal any significant differences in haemostatic factors that may explain this phenomenon.

Additionally, while hypothyroidism at a population level is likely to increase the risk of thromboembolic complications, its direct impact on haemostatic factor activities is ambiguous. Indicators of both hyper- and hypocoagulability have been reported, including prolonged half-life and decreased liver synthesis of haemostatic factors, respectively [40]. Indeed, studies on the coagulation system have suggested a reduction in fibrinolytic and endogenous anticoagulant activity, leading to prothrombotic tendencies, along with varied effects on certain coagulation factors—an increase in fibrinogen and factor VIII, but a decrease in factors VII, IX–XII [41]. Similarly, a divergent direction of changes in haemostatic factors has been observed in our NS group. Hypothyroidism was associated, on the one hand, with decreased factor X activity and, on the other hand, with a decrease in the protein S level. The consequences of these findings remain elusive and need to be the subject of further study.

### Pathomechanism of thyroid and haemostatic disorders in NS

It has been postulated that many complications of NS result from urinary leakage of various low molecular weight proteins, leading to their plasma deficiency, and conversely, liver oversynthesis and increased plasma levels of high molecular weight proteins. However, our results do not entirely support this hypothesis in the context of the development of both a hypercoagulable state and thyroid dysfunction. Instead, they suggest that the pathomechanisms of NS complications are more complex than previously expected.

Consistent with the main theory, we observed higher levels of high molecular weight factors in the NS group, including fibrinogen (340 kDa); factors V (330 kDa), VIII (330 kDa) and XI (160 kDa); von Willebrand factor (>500 kDa) and α2-macroglobulin (725 kDa). However, the directions of change in lower molecular weight factor levels were discordant. As expected, lower levels of certain low molecular weight proteins, such as antithrombin (55 kDa) and protein S (69 kDa), were noted, but, in contrast, increased levels of other low-size proteins were also found, including factors II (69 kDa) and VII (50 kDa), plasminogen activator inhibitor-1 (55 kDa) and α2-antiplasmin (70 kDa), as well as protein C (62 kDa). These discrepancies, reported previously [12, 31, 34], may be related to differences in half-life, rates of urinary excretion and different speeds of synthesis of these haemostatic proteins, although this remains speculative.

Similarly, the directions of changes in thyroid hormone carrier levels were inconsistent despite their low molecular weight, which should result in leakage into urine. Previous studies, which although small, demonstrated significant urinary loss of thyroxine-binding globulin, prealbumin, T3 and T4 [4–7]. However, we found the thyroxine-binding globulin concentration was higher in NS than in the control group. Furthermore, the correlation between TSH and thyroxine-binding globulin was positive, not negative, as expected. Additionally, higher thyroxine-binding globulin values were observed in hypothyroidism than in ESS.

Moreover, our results do not clearly follow the postulated continuum from euthyreosis, through ESS, to hypothyroidism along with increasing proteinuria. Unlike previous studies [11, 42, 43], we did not observe such clear relationships between parameters of thyroid function and proteinuria or carrier proteins. We did not find differences between euthyroid, ESS and hypothyroid patients in terms of NS severity, despite a moderate negative relationship between TSH and serum albumin. This suggests that the development of thyroid dysfunction in NS is not solely a consequence of urinary loss of hormones and their binding proteins. Potentially, changes in feedback regulation, synthesis and release rates, and the half-life of hormones and carrier proteins, together with haemodynamic changes associated with tissue oedema, which may alter hormone distribution volume and peripheral conversion, could play a pivotal role in the regulation of thyroid hormones [1]. In addition, these effects may be negatively influenced by the hypercatabolic state in NS, further altering various hormonal axes.

Overall, the pathophysiology of both coagulation and thyroid disorders in severe NS appears to be more complex than simply resulting from urinary leakage of proteins dependent on their molecular weight. However, the underlying processes have not been thoroughly investigated and therefore the exact mechanisms remain unclear.

### Study limitations

Although our study confirmed the common occurrence of thyroid dysfunction in NS and provided new insights into the complexity of haemostatic and thyroid disorders, it has several limitations. The small sample size, a lack of longitudinal follow-up and missing data on antithyroid peroxidase antibodies limit the conclusions that can be drawn. Specifically, findings regarding the relationship between ESS and low lean tissue mass and between hypothyroidism and coagulation disorders cannot be interpreted as causal. Large cohort studies are needed to further characterize the interplay between thyroid dysfunction in NS, nutrition and coagulation.

## CONCLUSIONS

This study demonstrated that thyroid dysfunction is a common phenomenon in severe NS, with ESS slightly predominating over hypothyroidism. Importantly, a substantial proportion of patients may require levothyroxine supplementation. These findings constitute an argument for routine thyroid function testing in this group of patients. The assessment of the interplay between thyroid dysfunction and nutritional or coagulation disorders requires further investigation.

## Supplementary Material

sfae280_Supplemental_Files

## Data Availability

The data underlying this article will be shared upon reasonable request to the corresponding author.
