# Supplementary material for: The impact of severe nephrotic syndrome on thyroid function, nutrition and coagulation
Source: Clin Kidney J. 2024 Sep 4;17(9):sfae280. doi: 10.1093/ckj/sfae280 (PMC11422718; doi:10.1093/ckj/sfae280)
Supplement: sfae280_Supplemental_Files [file sfae280_supplemental_files.zip › Supplementary Materials.pdf]

SUPPLEMENTARY MATERIALS

Table of contents

Table S1. Characteristics of patients with different histological diagnosis. 2

Table S2. Characteristics of patients with new diagnosis and relapse of nephrotic syndrome. 3

Table S3. Summary of indications for levothyroxine supplementation in patients in the NS group. 4

Table S4. Correlation matrix of thyroid hormones, carrier proteins, nutritional and coagulation parameters in severe nephrotic syndrome. 5

Table S5. Univariate and multivariate linear models evaluating the impact of thyroid function on lean tissue mass in severe nephrotic syndrome. 6

Table S6. Univariate and multivariate linear models evaluating the impact of thyroid function on factor X activity in severe nephrotic syndrome. 7

Table S7. Univariate and multivariate linear models evaluating the impact of thyroid function on protein S activity in severe nephrotic syndrome. 8

SUPPLEMENTARY MATERIALS

Table S1. Characteristics of patients with different histological diagnosis.

Variable	MCD/FSGS *(N = 22)*	MN *(N = 11)*	Other/Unknown *(N = 9)*	*P* – value
**Demography and anthropometry**				
Sex: Male, *n(%)*	13 (59.0%)	10 (90.9%)	6 (66.7%)	0.173
Female, *n(%)*	9 (45.0%)	1 (9.1%)	3 (33.3%)	
Age, *years*	38 ± 17	58 ± 14	63.8 ± 15	[a b] **< 0.001**
Height, *cm*	170 ± 11	172 ± 11	167 ± 9	0.504
Weight, *kg*	79.9 ± 17.5	83.8 ± 13.0	81.5 ± 19.0	0.819
**NS course**				
Serum albumin, *g/dL*	2.1 (1.6 – 2.3)	2.3 (1.6 – 2.4)	2.5 (2.0 – 2.5)	0.217
Proteinuria, *g/24h*	8.3 (6.1 – 11.5)	10.9 (6.3 – 14.7)	11.9 (4.7 – 14.9)	0.889
Duration of nephrotic syndrome, *months*	0.3 (0.2 – 0.8)	4.0 (1.0 – 12.0)	2.0 (1.0 – 12.0)	[a b] **< 0.001**
Serum creatinine, *mg/dL*	0.9 (0.7 – 1.3)	1.5 (0.8 – 1.9)	1.5 (1.4 – 1.7)	0.101
eGFR, *$mL/min/1.73m^2$*	85 (60 – 109)	52 (39 – 105)	51 (34 – 55)	0.063
**Prior history of thyroid disorder**				
Hashimoto disease, *n(%)*	1 (4.6%)	1 (9.1%)	2 (22.2%)	0.314
None, *n(%)*	21 (95.4%)	10 (90.9%)	7 (77.8%)	
**Newly diagnosed thyroid dysfunction**				
Hypothyroidism, *n(%)*	7 (31.8%)	1 (9.1%)	5 (55.6%)	
ESS, *n(%)*	8 (36.4%)	4 (36.4%)	3 (33.3%)	0.177
None, *n(%)*	7 (31.8%)	6 (54.5%)	1 (11.1%)	
**Thyroid hormones and carrier proteins**				
TSH, *μIU/mL*	2.96 (1.35 – 5.25)	2.05 (0.57 – 2.76)	4.37 (2.12 – 5.77)	0.129
fT4, *pmol/L*	12.48 (10.58 – 16.31)	12.33 (11.04 – 13.78)	15.42 (9.86 – 16.69)	0.926
fT3, *pmol/L*	3.13 (2.28 – 3.78)	3.56 (2.53 – 3.95)	3.34 (3.22 – 3.44)	0.885
Thyroxine-binding globulin, *ng/mL*	4.33 (2.69 – 5.25)	2.92 (2.58 – 3.91)	3.50 (2.88 – 3.89)	0.160
Prealbumin, *mg/dL*	27 (23 – 28)	30 (23 – 34)	21 (15 – 25)	[c] **0.034**
**Nutrition**				
Lean tissue mass, *kg*	38.2 ± 11.2	42.8 ± 11.3	33.3 ± 11.0	0.177
Adipose tissue mass, *kg*	33.5 (30.2 – 43.6)	33.6 (28.5 – 35.9)	43.8 (29.7 – 45.6)	0.633
Total cholesterol, *mg/dL*	372 (335 – 458)	325 (229 – 358)	290 (260 – 398)	0.050
LDL, *mg/dL*	263 (238 – 323)	210 (136 – 261)	215 (142 – 310)	0.066
Triglycerides, *mg/dL*	192 (128 – 249)	201 (167 – 387)	180 (128 – 334)	0.810
Total iron-binding capacity, *μg/dL*	172 ± 41	177 ± 43	180 ± 32	0.870
**Hemostasis**				
Fibrinogen, *mg/dL*	687 ± 163	625 ± 117	643 ± 288	0.639
Factor II, *%*	119 ± 17	117 ± 13	102 ± 20	[b] **0.047**
Factor V, *%*	138 ± 48	121 ± 48	106 ± 46	0.215
Factor VII, *%*	122 ± 37	110 ± 26	123 ± 33	0.552
Factor VIII, *%*	185 ± 75	140 ± 39	176 ± 73	0.200
Factor IX, *%*	160 ± 41	136 ± 32	140 ± 66	0.273
Factor X, *%*	100 ± 23	104 ± 24	96 ± 28	0.748
Factor XI, *%*	147 ± 37	114 ± 16	112 ± 41	[a b] **0.009**
Factor XII, *%*	78 ± 45	79 ± 24	72 ± 32	0.881
von Willebrand factor, *%*	120 (117 – 262)	218 (111 – 324)	193 (119 – 364)	0.539
Protein S, *%*	79 ± 23	90 ± 23	74 ± 21	0.251
Protein C, *%*	248 ± 80	209 ± 39	202 ± 53	0.138
Antithrombin, *%*	75 ± 24	86 ± 17	86 ± 18	0.307
Plasminogen, *%*	93 ± 16	103 ± 11	91 ± 14	0.127
PAI-1, *ng/mL*	1.06 (0.59 – 1.76)	0.73 (0.30 – 1.59)	0.69 (0.45 – 1.35)	0.417
α2-antiplasmin, *%*	116 ± 13	115 ± 11	105 ± 16	0.122
α2-macroglobulin, *mg/dL*	364 (305 – 422)	400 (280 – 622)	341 (272 – 449)	0.784

eGFR – estimated glomerular filtration rate; ESS – euthyroid sick syndrome; FSGS – focal segmental glomerulosclerosis; fT3 – free triiodothyronine; fT4 – free thyroxine; LDL – low density lipoprotein; MCD – minimal change disease; MN – membranous nephropathy; NA – not applicable; NS – nephrotic syndrome; Plasminogen activator inhibitor-1; TSH – thyroid-stimulating hormone.

Footnotes (a-c) indicate statistically significant results (P < 0.05) of the pairwise comparisons of the following groups: (a) MCD/FSGS vs MN; (b) MCD/FSGS vs Other/Unknown; (c) MN vs Other/Unknown.

SUPPLEMENTARY MATERIALS

Table S2. Characteristics of patients with new diagnosis and relapse of nephrotic syndrome.

Variable		New diagnosis of NS *(N = 23)*	Relapse of NS *(N = 19)*	*P* – value
**Demography and anthropometry**				
Sex:	Male, *n(%)*	16 (69.6%)	13 (68.4%)	0.999
	Female, *n(%)*	7 (30.4%)	6 (31.6%)	
Age, *years*		56 ± 17	40 ± 19	**0.007**
Height, *cm*		170 ± 11	169 ± 10	0.860
Weight, *kg*		81.2 ± 17.5	81.3 ± 15.7	0.986
**NS course**				
Serum albumin, *g/dL*		2.0 (1.6 – 2.4)	2.0 (2.1 – 2.4)	0.181
Proteinuria, *g/24h*		11.9 (6.0 – 15.8)	7.6 (5.3 – 9.4)	0.106
Duration of nephrotic syndrome, *months*		1.0 (0.8 – 12.0)	0.3 (0.2 – 2.0)	**0.023**
Histology:	MCD/FSGS, *n(%)*	8 (34.8%)	14 (73.7%)	
	MN, *n(%)*	7 (30.4%)	4 (21.0%)	**0.022**
	Other or unknown, *n(%)*	8 (34.8%)	1 (5.3%)	
Serum creatinine, *mg/dL*		1.3 (0.8 – 1.6)	1.1 (0.8 – 1.7)	0.960
eGFR, *mL/min/1.73m²*		68 (42 – 105)	82 (39 – 109)	0.686
**Prior history of thyroid disorder**				
Hashimoto disease, *n(%)*		3 (13.0%)	1 (5.3%)	0.613
None, *n(%)*		20 (87.0%)	18 (94.7%)	
**Newly diagnosed thyroid dysfunction**				
Hypothyroidism, *n(%)*		9 (39.1%)	4 (21.1%)	
ESS, *n(%)*		8 (34.8%)	7 (36.8%)	0.384
None, *n(%)*		6 (26.1%)	8 (42.1%)	
**Thyroid hormones and carrier proteins**				
TSH, *μlU/mL*		3.45 (2.05 – 5.02)	1.69 (0.52 – 3.70)	**0.033**
fT4, *pmol/L*		13.64 ± 4.11	12.52 ± 3.01	0.327
fT3, *pmol/L*		3.34 ± 0.87	3.16 ± 0.93	0.517
Thyroxine-binding globulin, *ng/mL*		3.75 ± 1.59	3.58 ± 1.79	0.749
Prealbumin, *mg/dL*		23 ± 7	28 ± 8	**0.042**
**Nutrition**				
Lean tissue mass, *kg*		37.5 ± 11.5	39.4 ± 11.5	0.594
Adipose tissue mass, *kg*		33.6 (28.7 – 43.9)	34.8 (28.5 – 45.1)	0.909
Total cholesterol, *mg/dL*		355 (281 – 441)	347 (260 – 453)	0.990
LDL, *mg/dL*		253 (185 – 327)	240 (173 – 318)	0.940
Triglycerides, *mg/dL*		183 (161 – 253)	201 (125 – 345)	0.840
Total iron-binding capacity, *μg/dL*		166 ± 33	187 ± 42	0.077
**Hemostasis**				
Fibrinogen, *mg/dL*		676 ± 207	643 ± 156	0.572
Factor II, *%*		111 ± 18	119 ± 17	0.169
Factor V, *%*		125 ± 50	128 ± 47	0.845
Factor VII, *%*		116 ± 33	123 ± 35	0.530
Factor VIII, *%*		163 ± 62	180 ± 76	0.432
Factor IX, *%*		138 ± 44	163 ± 45	0.081
Factor X, *%*		92 ± 19	110 ± 26	**0.011**
Factor XI, *%*		120 ± 44	144 ± 24	**0.038**
Factor XII, *%*		67 ± 33	90 ± 39	**0.041**
von Willebrand factor, *%*		119 (114 – 297)	163 (118 – 288)	0.464
Protein S, *%*		74 ± 23	90 ± 18	0.021
Protein C, *%*		221 ± 63	236 ± 74	0.506
Antithrombin, *%*		80 ± 17	81 ± 27	0.837
Plasminogen, *%*		95 ± 13	96 ± 17	0.826
PAI-1, *ng/mL*		1.02 ± 0.69	1.20 ±0.89	0.468
α2-antiplasmin, *%*		111 ± 14	116 ± 13	0.277
α2-macroglobulin, *mg/dL*		411 ± 152	356 ± 98	0.180

eGFR – estimated glomerular filtration rate; ESS – euthyroid sick syndrome; FSGS – focal segmental glomerulosclerosis; fT3 – free triiodothyronine; fT4 – free thyroxine; LDL – low density lipoprotein; MCD – minimal change disease; MN – membranous nephropathy; NA – not applicable; NS – nephrotic syndrome; Plasminogen activator inhibitor-1; TSH – thyroid-stimulating hormone.

SUPPLEMENTARY MATERIALS

Table S3. Summary of indications for levothyroxine supplementation in patients in the NS group.

**No**	**Age** [years]	**Sex**	**Prior thyroid disease**	**Prior levothyroxine dose** [μg]	**Recommended levothyroxine dose** [μg]	**TSH** [uIU/mL] *(reference: 0.27-4.20)*	**fT4** [pmol/L] *(reference: 12-22)*	**fT3** [pmol/L] *(reference: 3.2-6.9)*	**Justification of the decision to supplement LT-4**
1	21	M	no	no	75	6.07	9.2	2.96	New overt hypothyroidism
2	22	M	no	no	50	10.09	10.96	3.00	New overt hypothyroidism
3	29	M	no	no	50	5.72	7.3	2.05	New overt hypothyroidism
4	30	M	no	no	25	5.25	10.49	3.87	New overt hypothyroidism
5	59	M	no	no	75	9.9	10.49	4.16	New overt hypothyroidism
6	80	F	no	no	75	28.63	9.33	3.44	New overt hypothyroidism
7	21	F	no	no	25	5.88	14.36	3.48	Subclinical hypothyroidism with severe hyperlipidemia (LDL 261 mg/dL)
8	31	F	no	no	50	6.44	16.61	4.24	Subclinical hypothyroidism with severe hyperlipidemia (LDL 310 mg/dL)
9	72	M	no	no	25	5.77	16.94	3.22	Subclinical hypothyroidism with very high cardiovascular risk
10	63	M	yes	75	100	4.96	11.11	2.53	Prior hypothyroidism due to Hashimoto disease – increasing the dose of LT-4 to achieve the therapeutic goal
11	74	F	yes	50	75	5.02	10.38	3.37	Prior hypothyroidism due to Hashimoto disease – increasing the dose of LT-4 to achieve the therapeutic goal

LT-4 – levothyroxine.

SUPPLEMENTARY MATERIALS

Table S4. Correlation matrix of thyroid hormones, carrier proteins, nutritional and coagulation parameters in severe nephrotic syndrome.

Correlation matrix is provided in the attached file (**Supplementary Table S2.xlsx**).

Associations between variables were assessed using Spearman rank correlation and presented with correlation coefficient (R) and p – value. P – values < 0.05 were considered statistically significant.

SUPPLEMENTARY MATERIALS

Table S5. Univariate and multivariate linear models evaluating the impact of thyroid function on lean tissue mass in severe nephrotic syndrome.

	Univariate models		Multivariate model		
**Variable**	**β (95% CI)**	***P* – value**	**Coefficient ± SE**	**β (95% CI)**	***P* – value**
**Thyroid function:**					
**Hypothyroidism**	-0.190 (-0.530 to 0.151)	0.266	-1.463 ± 1.430	-0.104 (-0.311 to 0.103)	0.313
**ESS**	-0.289 (-0.629 to 0.051)	0.094	-2.826 ± 1.381	-0.209 (-0.416 to -0.002)	**0.048**
**Euthyroidism**	*ref.*		*ref.*		
**Age**, *years*	-0.319 (-0.622 to -0.017)	**0.039**	-0.117 ± 0.056	-0.200 (-0.393 to -0.007)	**0.043**
**Sex:**					
**Male**	0.683 (0.450 to 0.917)	**< 0.001**	5.258 ± 1.430	0.432 (0.194 to 0.671)	**0.001**
**Female**	*ref.*		*ref.*		
**Serum albumin**, *g/dL*	-0.024 (-0.344 to 0.295)	0.879	–	–	–
**Proteinuria**, *g/24h*	0.237 (-0.073 to 0.547)	0.131	–	–	–
**eGFR,** *mL/min/1.73m²*	0.058 (-0.261 to 0.377)	0.717	–	–	–
**Height**, *cm*	0.736 (0.52 to 0.952)	**< 0.001**	0.425 ± 0.141	0.387 (0.127 to 0.648)	**0.005**
**Weight**, *kg*	0.431 (0.142 to 0.719)	**0.004**	-0.035 ± 0.075	-0.051 (-0.273 to 0.171)	0.643
**Intercept**	–	–	-27.187 ± 23.300	–	0.251
**Assumptions:**					
P – value of Shapiro-Wilk test for normal distribution of residuals testing				0.773	
**Quality of the models:**					
		Corrected R^2		0.74	

ESS – Euthyroid sick syndrome; eGFR – estimated glomerular filtration rate; β – standardized coefficient; R^2 – coefficient of determination.

SUPPLEMENTARY MATERIALS

Table S6. Univariate and multivariate linear models evaluating the impact of thyroid function on factor X activity in severe nephrotic syndrome.

	Univariate models		Multivariate model		
**Variable**	**β (95% CI)**	***P* – value**	**Coefficient ± SE**	**β (95% CI)**	***P* – value**
**Thyroid function:**					
**Hypothyroidism**	-0.520 (-0.844 to -0.196)	**0.002**	-12.771 ± 4.602	-0.430 (-0.744 to -0.116)	**0.009**
**ESS**	0.038 (-0.286 to 0.362)	0.813	1.087 ± 4.316	0.038 (-0.267 to 0.343)	0.803
**Euthyroidism**	*ref.*		*ref.*		
**Serum albumin**, *g/dL*	0.443 (0.156 to 0.729)	**0.003**	20.073 ± 8.182	0.322 (0.058 to 0.606)	**0.019**
**Proteinuria**, *g/24h*	-0.026 (-0.346 to 0.293)	0.868	–	–	–
**eGFR**, ***m****L/min/1.73m*^{2}	-0.007 (-0.327 to 0.312)	0.964	–	–	–
**Intercept**	–	–	58.871 ± 16.976	–	0.001
**Assumptions:**					
P – value of Shapiro-Wilk test for normal distribution of residuals testing				0.387	
**Quality of the models:**					
		Corrected R^2		0.35	

ESS – Euthyroid sick syndrome; eGFR – estimated glomerular filtration rate; β – standardized coefficient; R^2 *– coefficient of determination.*

SUPPLEMENTARY MATERIALS

Table S7. Univariate and multivariate linear models evaluating the impact of thyroid function on protein S activity in severe nephrotic syndrome.

	Univariate models		Multivariate model		
**Variable**	**β (95% CI)**	***P* – value**	**Coefficient ± SE**	**β (95% CI)**	***P* – value**
**Thyroid function:**					
**Hypothyroidism**	-0.448 (-0.772 to -0.124)	**0.007**	-11.105 ± 4.503	-0.405 (-0.737 to -0.073)	**0.018**
**ESS**	-0.092 (-0.416 to 0.232)	0.570	-2.432 ± 4.223	-0.092 (-0.415 to 0.231)	0.568
**Euthyroidism**	*ref.*		*ref.*		
**Serum albumin**, *g/dL*	0.281 (-0.024 to 0.586)	0.070	8.980 ± 8.007	0.161 (-0.129 to 0.451)	0.269
**Proteinuria**, *g/24h*	-0.212 (-0.525 to 0.100)	0.177	–	–	–
**eGFR**, *mL/min/1.73m*^{2}	-0.182 (-0.496 to 0.132)	0.249	–	–	–
**Intercept**			62.026 ± 16.613	–	0.001
**Assumptions:**					
P – value of Shapiro-Wilk test for normal distribution of residuals testing				0.772	
**Quality of the models:**					
Corrected R^2				0.27	

ESS – Euthyroid sick syndrome; eGFR – estimated glomerular filtration rate; β – standardized coefficient; R^2 *– coefficient of determination.*
